# Fabrication and characterization of hybrid thermoelectric materials based on aligned nanowires

**DOI:** 10.3389/fchem.2024.1407129

**Published:** 2024-09-26

**Authors:** Min-Jeong Lee, Chae Yoon Kim, Jae-Hong Lim

**Affiliations:** Department of Material Science and Engineering, Gachon University, Seongnam, Republic of Korea

**Keywords:** thermoelectrics, hybrid thermoelectrics, te nanowire, galvanic displacement reaction, topotactic reaction

## Abstract

This study introduces the synthesis of a hybrid thermoelectric material with enhanced conductivity and a high Seebeck coefficient, leveraging the properties of Te nanowires (NWs) and the conductive polymer PEDOT:PSS. Te NWs were synthesized using the galvanic displacement reaction. To further enhance conductivity, Ag-Te NWs were synthesized under optimized conditions via the Ag topotactic reaction, achieving desired results within 7 min using ethylene glycol and AgNO_3_. This hybrid material exhibited an electrical conductivity of 463 S/cm, a Seebeck coefficient of 69.5 μV/K at 300 K, and a power factor of 260 μW/mK^2^. These metrics surpassed those of conventional Te/PEDOT:PSS hybrids by a factor of 3.6, highlighting the superior performance of our approach. This study represents a significant advancement in thermoelectric materials, improving both conductivity and efficiency.

## 1 Introduction

In recent years, global climate change has worsened because of the increasing carbon emissions caused by the combustion of fossil fuels. This has necessitated the exploration of sustainable alternatives for energy generation. Consequently, many alternative energy sources have been actively developed. Among these, thermoelectric power generation emerges as a promising method, harnessing waste heat by using thermoelectric generators. Considering the substantial amount of unused waste heat generated by car exhausts and during industrial processes, there is a pressing need for technologies that can easily convert heat to electricity. To this end, there has been a surge in research efforts aimed at integrating thermoelectric elements into semiconductors, home appliances, power generation systems, aerospace devices, and other industrial applications. However, the seamless integration into these applications necessitates efficiency enhancement (Toberer, 2008; Chen et al., 2019; Wei et al., 2020; Yuan et al., 2021).

Thermoelectricity is a phenomenon wherein thermal energy and electrical energy are directly and reversibly converted based on the movement of electrons or holes on the basis of a temperature difference within a solid material. In this phenomenon, the voltage due to the temperature difference between the junction and the opposite side generates thermal power, and current flows in the closed circuit. This is the basic principle in thermoelectric power generation, and it involves the conversion of heat to electricity, also referred to as the Seebeck effect. The performance is expressed as ZT and is determined using the following equation:

$$ZT=\frac{S\sigma^{2}T}{\kappa },$$

where S, σ, T, and κ are the Seebeck coefficient, electrical conductivity, absolute temperature, and thermal conductivity, respectively. In this equation, Sσ^2^ can be expressed as a power factor (PF) (Chen et al., 2019; Bell, 2008; Shi et al., 2020).

The thermoelectric performance can be improved by increasing the Seebeck coefficient value and electrical conductivity. However, controlling these parameters independently poses challenges owing to their interrelation. Thus, for independent operations, we chose nanoscale-diameter wires. The large surface area of Te nanowires (NWs) helps suppress the decrease in thermal conductivity due to phonon scattering (Chen et al., 2019). In the case of electrical conductivity, the impact of the surface effect is negligible. Furthermore, the Seebeck coefficient can be increased via carrier energy filtering or quantum confinement effects (Chen et al., 2019; Wang et al., 2013; Mao et al., 2016).

A previous study reveals that compared with group IV and III-V semiconductors, Te exhibits low lattice thermal conductivity owing to its relatively high atomic weight and crystal structure (Gao et al., 2018). Te, a key semiconductor element, has a Seebeck coefficient of approximately 700 μV/K at room temperature (Goldsmid and Gray, 1979; Lin et al., 2016). Despite its high Seebeck coefficient, the independent use of Te in thermoelectric devices is challenging primarily because of its low electrical conductivity (100 S/cm), which reduces the PF. Reacting metal ions with Te to form telluride can effectively increase electrical conductivity, thereby enhancing thermoelectric properties (Zeng et al., 2019). The thermoelectric performance of Te NWs, which have an electrical conductivity and a Seebeck coefficient of 10 S/m and 400 μV/K, respectively (Wang et al., 2018), can be improved by forming composites with Ag. The high electrical conductivity of Ag (6.2 × 10^7 S/m) compensates for the conductivity deficiency of Te. The resulting Ag-Te composites, formed through a topotactic reaction (Zhang et al., 2011), not only exhibit reduced thermal conductivity due to the disordered structure of Ag atoms within the Ag-Te lattice but also demonstrate increased electrical conductivity. The enhanced electron mobility significantly increases the PF of Ag-Te composites (Gao et al., 2018; Zeng et al., 2019; Ishiwata et al., 2013; Wang et al., 2019; Mazzio et al., 2020).

Despite possessing a high PF, further improvements in properties are necessary for the commercialization of these composites. To this end, organic and inorganic hybrid thermoelectric materials were synthesized using Ag-Te/poly (3,4-ethylenedioxythiophene) polystyrene sulfonate (PEDOT:PSS), which exhibits favorable properties (Zhang et al., 2010; Kim et al., 2013; Shi et al., 2015; Russ et al., 2016; Song and Cai, 2017). PEDOT, a conductive polymer, is widely used in thermoelectric applications due to its excellent electrical conductivity, flexibility, and solution-processability. These properties make it an ideal candidate for forming composites with inorganic materials like Te NWs. Aligned NWs optimize the path of charge carriers, significantly enhancing electrical conductivity. This structural configuration allows electrons and holes to move more efficiently along the NWs, thereby increasing overall thermoelectric efficiency. As the NWs are arranged in a consistent direction, thermal conductivity is reduced because this arrangement limits the transmission of heat perpendicular to the direction of the NWs. The performance of thermoelectric materials is optimized by the combination of low thermal conductivity and high electrical conductivity (Hangarter and Myung, 2005; Hasan et al., 2022).

Previous studies have highlighted the potential of thermoelectric materials but have faced limitations in achieving high electrical conductivity and low thermal conductivity simultaneously. The challenge remains in effectively integrating these materials for practical applications. This study addresses a gap in the existing literature by focusing on the fabrication and characterization of hybrid thermoelectric materials based on aligned nanowires, which optimize electron transport and minimize thermal conductivity, thereby enhancing overall thermoelectric performance.

In this study, we focused on enhancing the electrical performance of thermoelectric materials using aligned silver telluride (Ag-Te) nanowires, known for their high electron mobility crucial for thermoelectric performance improvement. Utilizing nanostructured Ag-Te optimized the electron behavior, thereby enhancing the overall efficiency of the materials. Integration with PEDOT, a polymer with excellent electrical conductivity, flexibility, and solution-processability, further improved the overall properties of the materials. This combination leverages the high conductivity of Ag-Te and flexibility of PEDOT, creating a composite that maintains high thermoelectric efficiency while being mechanically robust and flexible. Hangarter and Myung (Hangarter and Myung, 2005; Hasan et al., 2022) discussed the advantages of aligned nanowires in optimizing charge-carrier paths, which enhance electrical conductivity by reducing scattering and allowing for more efficient electron transport. Additionally, Hasan et al. (2022) highlighted the benefits of integrating nanostructured materials with polymers to improve mechanical properties without significantly compromising electrical performance. These studies provide a foundational basis for our approach, demonstrating that the strategic combination of nanostructured materials and polymers can yield composites having superior thermoelectric and mechanical properties. This study is significant in that it combines the high electrical conductivity of Ag-Te with the flexibility and processability of PEDOT to create a mechanically robust hybrid thermoelectric material. To our knowledge, this is one of the first studies to utilize aligned Ag-Te nanowires in combination with PEDOT to achieve enhanced thermoelectric performance, addressing a notable gap in the existing literature.

By adhering to this structure, the introduction section will provide a clear and concise overview of the study’s purpose and methodology, setting the stage for a more focused and coherent discussion of results in the relevant sections.

## 2 Materials and methods

### 2.1 Materials

p-Type boron-doped Si (100) wafer (1–10 Ω·cm, 525 μm), cadmium chloride (CdCl_2_, 99.0% min), and polyvinylidene fluoride (PVDF, powder) were procured from Alfa Aesar. Hydrofluoric acid (HF, 49.0%), silver nitrate (AgNO_3_, 99.8%), isopropyl alcohol (IPA, anhydrous 99.5%), and ethylene glycol (EG, 99.5%) were bought from Samchun Chemical Co., Ltd. Tellurium dioxide (TeO_2_, 99.0%) and N-methyl-2-pyrrolidone (NMP, 99.5%) were obtained from Sigma Aldrich company. PEDOT:PSS (Clevios PH 1000) was obtained from the Heraeus company.

### 2.2 Synthesis of Ag-Te NWs

The Te NWs were grown through a galvanic displacement reaction, which is an electrochemical reaction induced by a difference in redox potential. This is a simple approach to selectively change the composition or structure of a nanomaterial based on the chemical transformation between a solid metal and metal ion. Unlike electrodeposition, galvanic displacement reactions do not require an external power supply, facilitating the construction of complex nanostructures. Furthermore, instantaneous nucleation induces the uniform formation of aligned Te NWs (Jung et al., 2010; Moon et al., 2011; Jeong et al., 2013).

In the synthesis of the aligned Te NWs, a Si wafer (2 × 2 cm) was used as a substrate. The Si substrate was first cleaned using acetone and dried using N_2_ gas. Next, it was washed with ethanol and again dried using N_2_ gas. The substrate was etched in 10% HF for 5 min to remove the oxide layer, followed by rinsing with deionized (DI) water and subjected to three ethanol washes before a final drying step with N_2_ gas. The solution was prepared by sequentially dissolving CdCl_2_ (1M), HF (4.5M), and TeO_2_ (1 mM) in DI water and stored in an oven pre-heated to 50°C. A HALOGEN DISPLAY/OPTIC lamp (Osram 64637; 12 V, 100 W) was used as the light source. Once the temperature of the solution reached 50°C, the silicon substrate was immersed and placed in the oven under light exposure for 72 h. Following synthesis, the aligned Te NWs, which were prone to detachment from the substrate, underwent multiple washes with DI water, followed by a final wash with IPA. Owing to their light sensitivity, the NWs were wrapped in aluminum foil to prevent light exposure and stored in an oven at 70°C for drying. A topotactic reaction solution was prepared by mixing 0.4 g of AgNO_3_ with 20 mL of EG and stirred at 200 rpm in 25°C for 30 min. After adding the Si/Te NWs to the solution and allowing them to react for 7 min, they were washed several times with ethanol and dried in an oven at 70°C. The as-synthesized Ag-Te NWs were covered with aluminum foil before storage to prevent light exposure.

### 2.3 Synthesis of Ag-Te NWs/PEDOT:PSS hybrid thermoelectric material

After mixing PEDOT:PSS and EG at a volume ratio of 85:15, 100 μL of the mixture was evenly applied to a 2 cm × 2 cm area of the as-synthesized Ag-Te NWs on the Si wafer substrate. The composite was then stored in a vacuum oven at 50°C for 17 h to ensure that it was well coated and dried. Subsequently, a solution of 50 mL of NMP and 2.5 g of PVDF was applied, and the NWs were stored in an oven at 50°C for 7 h. The dried Ag-Te NWs/PEDOT:PSS/PVDF film was peeled off from the Si wafer.

### 2.4 Characterization of Te NWs, Ag-Te NWs, and Ag-Te NWs/PEDOT:PSS

The morphology of growth of Te NWs was examined by scanning electron microscopy (SEM; HITACHI S-4300) and transmission electron microscopy (TEM; FEI Tecnai) at an accelerating voltage of 300 kV. Moreover, for analyzing the crystal structures of the samples, X-ray diffraction (XRD) was conducted at 2θ = 15°–60° (Rigaku SmartLab) at a scan rate of 2°/min using Cu Kα radiation (λ = 1.5406 Å). The chemical state was confirmed through X-ray photoelectron spectrometry (XPS; Thermo Scientific Nexsa G2). To measure the work function, ultraviolet photoelectron spectroscopy (UPS; Thermo VG Scientific) was performed. A Keithley 2400 electrometer was used to measure the electrical conductivity of the synthesized Ag-Te NWs and Ag-Te NWs/PEDOT:PSS using a four-point probe technique. The Seebeck coefficient was measured using an in-house equipment. The Seebeck coefficient was determined from the plots of the measured Seebeck voltages as a function of the temperature difference (<2°C) across the specimen (S = ΔV/ΔT) (Kim et al., 2015). Nine samples were analyzed under each condition, and each measurement was repeated five times.

## 3 Results and discussion

When HF is included in the galvanic displacement reaction, galvanic displacement to Si occurs at the surface where both anodic and cathodic processes occur, indicating the possibility of redox reactions through the substrate. Fluoride ions in the solution react with the Si substrate and change it to silicon hexafluoride. As a result, the new surface of the silicon substrate is continuously exposed, which ensures a fresh reactive surface for the ongoing displacement reactions. This continuous exposure of fresh Si surfaces helps maintain a consistent reaction rate by preventing the passivation of the surface and ensuring ongoing redox activity (Carraro et al., 2007; Park et al., 2013; Kok et al., 2018). In general, the overall reaction equation of the redox couple is as follows (Equation 1):

$${Si}^{0}\left(s\right)+{6F}^{-}\left(\text{aq}\right)\to {{\text{SiF}}_{6}}^{2-}\left(\text{aq}\right)+4e^{-}\;E_{0}=-{1.20\;V}_{\text{SHE}}$$ (1)

$${{\text{HTeO}}_{2}}^{+}\left(\text{aq}\right)+{3H}^{+}\left(\text{aq}\right)+{4e}^{-}\;\to {\text{Te}}^{0}\left(s\right)+2H_{2}O\left(l\right)\;E_{0}={0.309\;V}_{\text{SHE}}$$ (2)

Because of the difference in the redox potentials of SiF_6_^2-^/Si^0^ (E_0_ = −1.20 V_SHE_) and HTeO_2_^+^/Te^0^ (E_0_ = 0.309 V_SHE_), a galvanic displacement reaction occurs (Jeong et al., 2013; Rudnik and Sobesto, 2011). The Wulff facet theorem for the shape control of crystal growth states that the relative surface energy of each crystal facet governs the formation of the crystal shape, and the structure with the minimum total surface energy is preferred. The symmetry of the elementary crystal lattice is reflected in the shape of the single crystal nanostructure. As Te possesses a unique helical structure, one-dimensional crystal structure growth occurs along the long c-axis (Mohanty et al., 2006).

**FIGURE 1:**
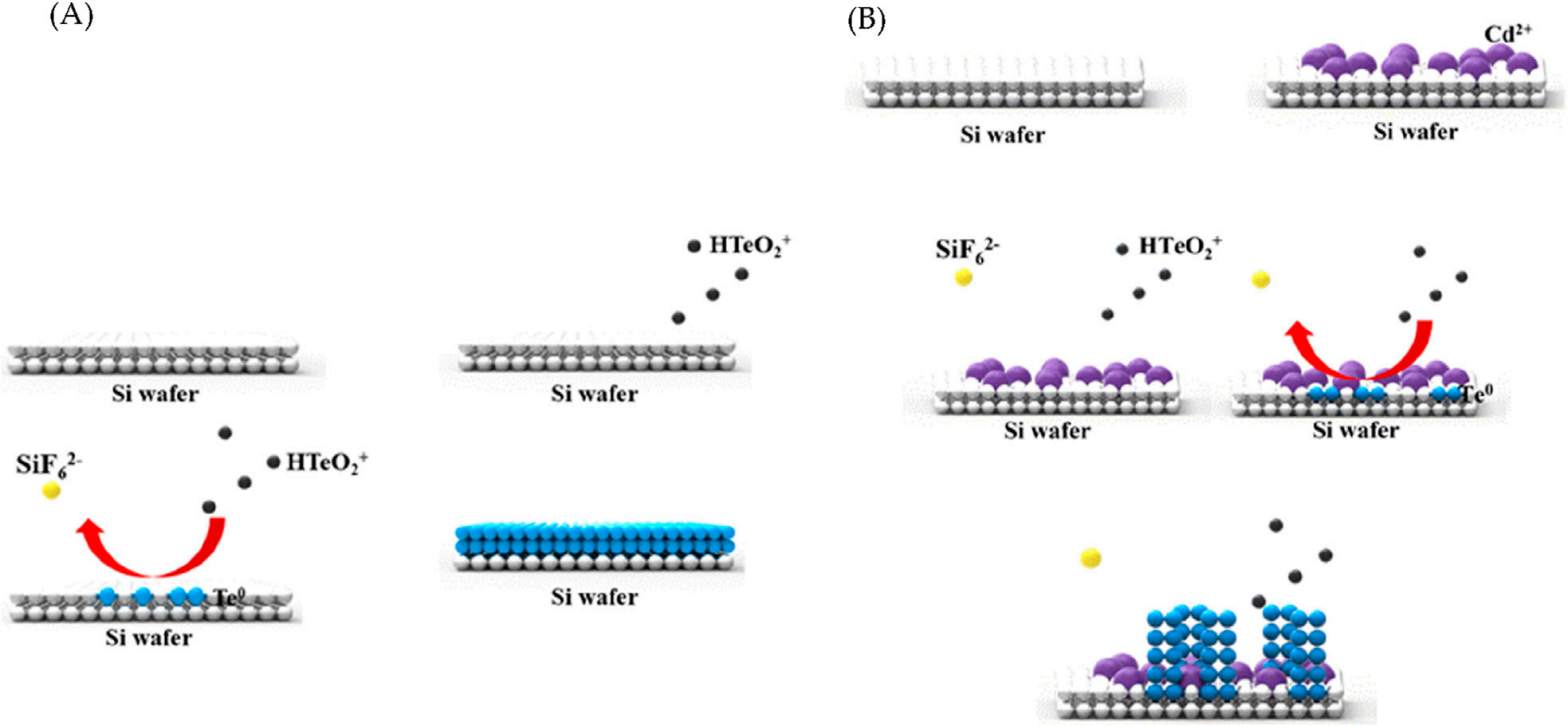
Schematics of the **(A)** galvanic displacement reaction of Te and **(B)** role of Cd^2+^ in the synthesis of Te nanowires (NWs).

Film growth is achieved through the Volmer–Weber growth method via microstructures (Floro et al., 2002; Chen et al., 2002). Initially, the nuclei of different crystallographic quadrilaterals are generated during the Volmer–Weber growth; these nuclei are then attached to each other by the expansion of additional islands. The islands continue to grow until the substrate is covered, after which the film thickens. Due to the growth mode, Te grows in the form of a film during the galvanic displacement reaction (Figure 1A). However, when Cd is added during synthesis, it interferes with the bonding among the nuclei and ensures the constant formation of islands and not a film. While maintaining the shape of an island, the process of attaching Te to the upper surface is repeated, after which it grows into a wire shape (Figure 1B).

Jeong et al. (Jeong et al., 2013) conducted a study on the time-dependent nucleation and growth of Te NWs on (100)-oriented Si substrates. The intensity of the (003) and (102) peaks increased after 24 h, indicating a preference for growth along the (003) direction. The final crystal shape was confirmed to be affected by the plane with the minimum surface free energy.

In the case of tetragonal Te (t-Te), the crystal structure exhibited significant anisotropy, exhibiting a distinct tendency for one-dimensional growth along the c-axis due to elongated growth. The Te seed layer exclusively formed on the surface without the attached Cd ions. Te demonstrated a tendency to grow into a helical structure (101), initiating a partial reaction that led to the formation of aligned wires (Figure 1B).

**FIGURE 2:**
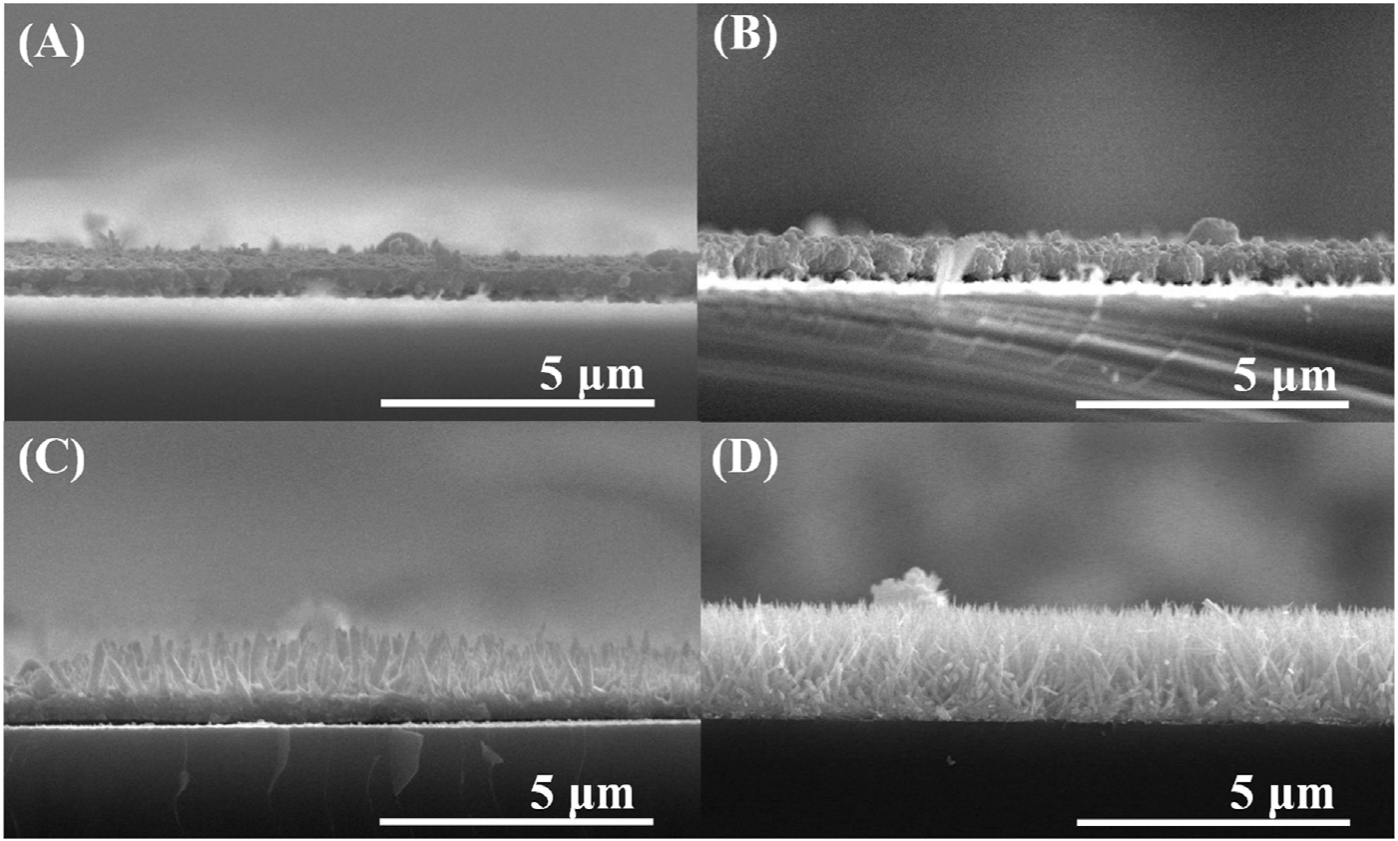
SEM images of Te NWs formed at Cd concentrations of **(A)** 0.001 **(B)** 0.01 **(C)** 0.1, and **(D)** 1 M.

Furthermore, the SEM images were analyzed to observe the synthesis of Te with respect to Cd concentration. Figure 2 illustrates the growth of Te NWs based on Cd concentration. Figure 2A, B indicate that the synthesized form appears as a film without significant differences. However, Te NWs of a diameter of 150 nm and lengths of up to 1 μm were formed at 0.1M (Figure 2C). At 1M, the needle-shaped aligned wires with a diameter of 10 nm and lengths of 2.5 μm were obtained (Figure 2D). This confirms that as the Cd concentration increases, aligned NWs are synthesized. Moreover, when the Cd concentration was below 0.1M, Te was synthesized in the form of a film. Thus, 1M was considered as the optimal concentration in this study.

**FIGURE 3:**
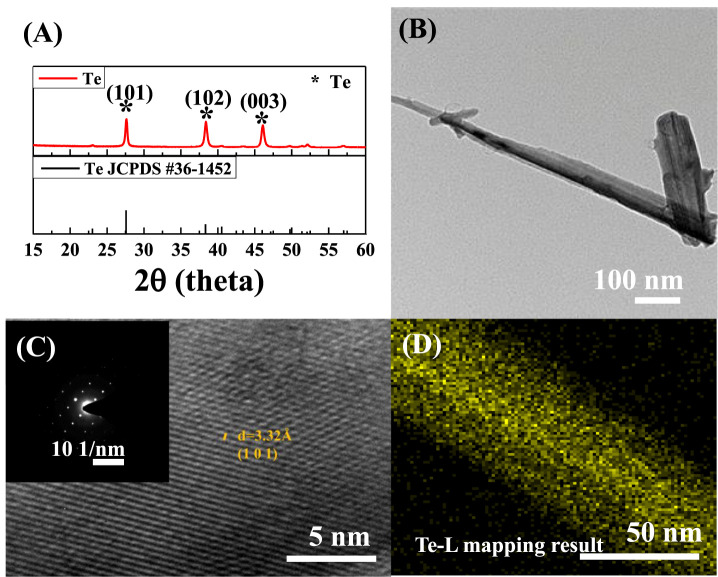
Te NWs synthesized using 1 M Cd **(A)** X-ray diffraction (XRD) pattern **(B)** transmission electron microscopy (TEM) image **(C)** high-resolution transmission electron microscopy (HR-TEM) image, selected area electron diffraction (SAED) pattern, and **(D)** EDX mapping profile.

Figure 3 shows the XRD patterns and TEM images, which confirm the structure and morphology of the Te NWs synthesized under the optimal conditions (Cd 1M). The XRD patterns of the as-synthesized Te NWs, shown in Figure 3A, confirm that the presence of the Te peak corresponding to the crystalline material, in accordance with JCPDS #361452. The approximate length of the Te NWs was 1 μm, and the diameter was 100 nm (Figure 3B). Figure 3C displays a high-resolution transmission electron microscopy (HR-TEM) image and selected area electron diffraction (SAED) pattern of a Te NW. The interplanar distance is 3.32 Å, which corresponds to the (101) lattice plane of Te. No dislocations or defects were observed on the NW, suggesting the presence of a single-crystal structure. In addition, the sample was analyzed through EDX; the results confirmed that no element other than Te was present (Figure 3D). Moreover, the results revealed that during the synthesis through the galvanic displacement reaction, without the interference of Cd ions, Te grows evenly in the form of a film (Figure 3A). However, when an appropriate number of Cd ions enter, the ions attach to the Si wafer surface, which deaccelerates the galvanic exchange reaction (Mohanty et al., 2006).

To improve the electrical conductivity of the previously synthesized Te NWs, Ag was doped with these NWs. The Ag-Te NWs were synthesized using a topotactic reaction with Ag. Although various ratios of Ag-Te could be used, the synthesis was conducted with Ag_2_Te as the target because Ag_2_Te serves as a narrow-bandgap semiconductor with high electron mobility and low lattice thermal conductivity through the intercalation of Ag between Te lattice structures (Chen et al., 2002). In addition, bulk Ag_2_Te is known to induce a thermoelectric effect (Gnanadurai et al., 2002). The structure and chemical composition of the synthesized Ag-Te NWs were analyzed through XRD and XPS, with the reaction times of 3, 5, 7, and 10 min in the topotactic solution for the Ag_2_Te synthesis.

**FIGURE 4:**
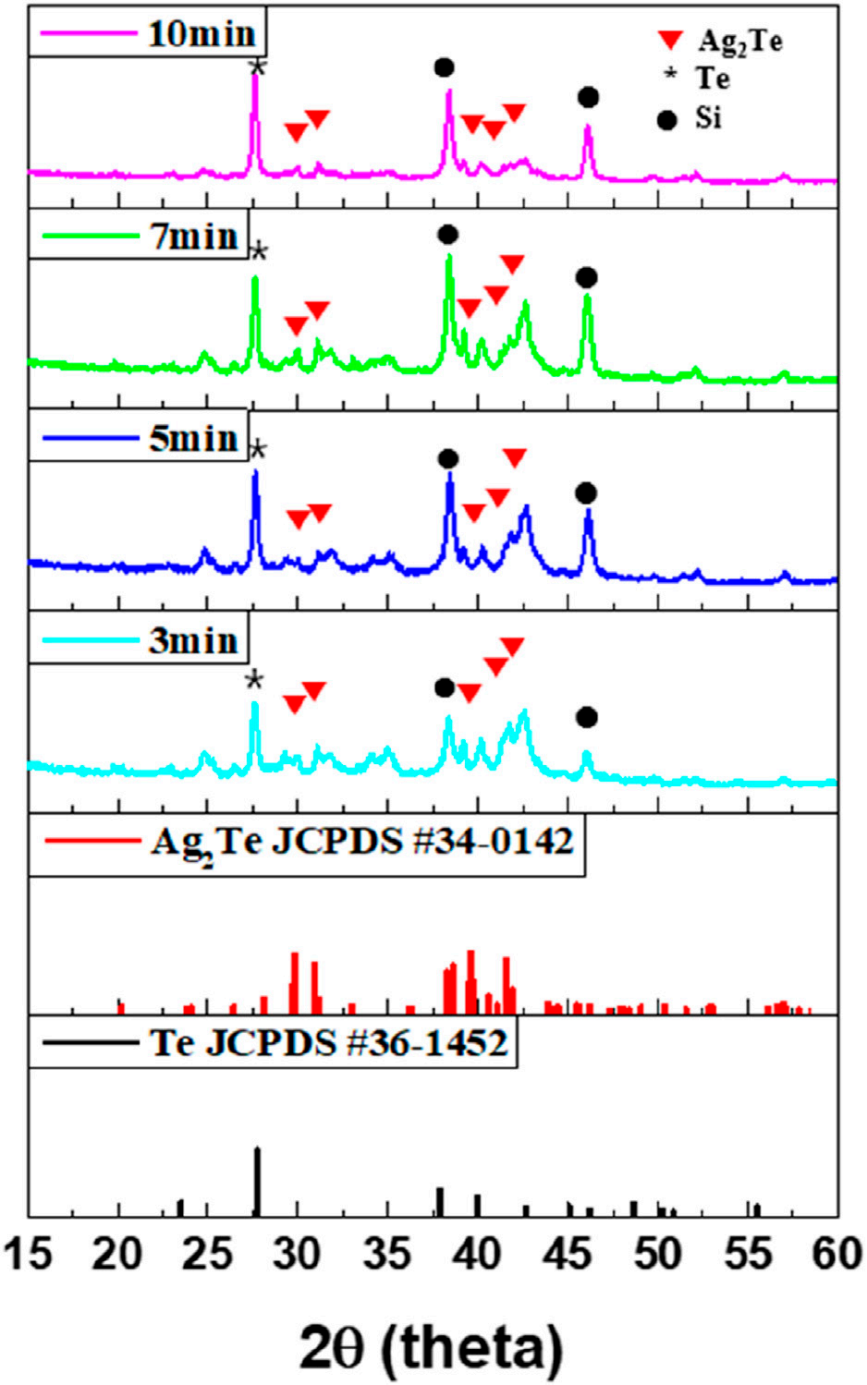
XRD patterns of Ag-Te at different topotactic reaction times.

The XRD patterns in Figure 4 indicate that the Ag_2_Te peak (JCPDS #340142) is present at all reaction times after 3 min. From 3 to 7 min, the intensity of the Ag_2_Te peak gradually increases, whereas at 10 min, it decreases. The peak changes at 10 min primarily because excess Ag is inserted between the Te structures and the helical structure of Te swells during the topotactic reaction. The swelling phenomenon causes the Te structures to expand, which, in turn, exposes the underlying Si wafer substrate as the reaction time increases. The interface between Te and Si could be the compound of Si-Te; however, it is amorphous (Lim et al., 2014). A possible formation mechanism of Ag_2_Te was proposed by Xiao et al. (2010). They reported that Ag ions could catalyze the disproportionate reaction of Te^0^ to Te^2-^ and Te^4+^ when Ag cations diffuse into Te. This can be defined as a topotactic reaction. Te^2-^ then combines with Ag^+^ to produce Ag_2_Te with a single-crystal structure. Consequently, Ag_2_Te is successfully synthesized by utilizing the topotactic reaction of Te in the aqueous solution at room temperature. Through this mechanism, Ag ions permeate into the Te NWs and the Ag_2_Te phase is formed in the Te matrix on the surface. However, the presence of the Te peak indicates that not all Te is converted to Ag_2_Te, resulting in the formation of a composite material containing both Te and Ag_2_Te phases. This dual-phase composition suggests that the reaction time and conditions were not sufficient to achieve the complete conversion of Te to Ag_2_Te.

**FIGURE 5:**
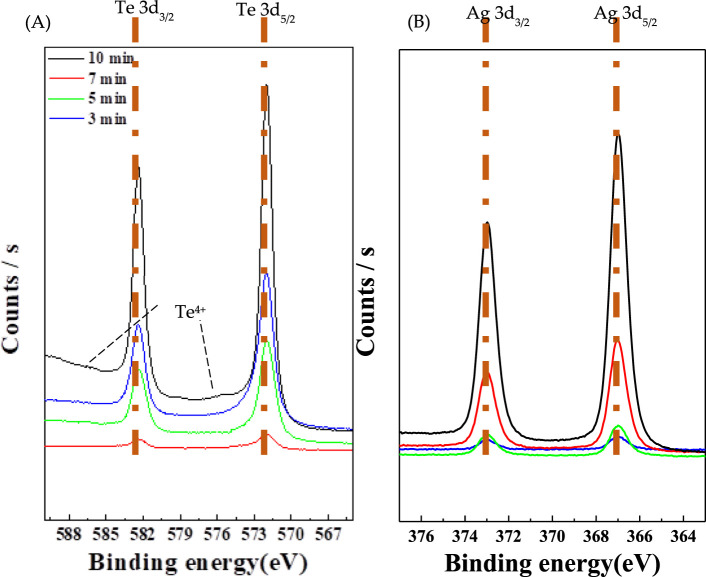
X-ray photoelectron spectra at different topotactic reaction times **(A)** Te and **(B)** Ag.

Figure 5 shows the XP spectra at differnet reaction times (3, 5, 7, and 10 min), confirming the presence of the Ag_2_Te phase. Figure 5A shows the Te 3d_3/2_ and Te 3d_5/2_ peaks, while Figure 5B shows the Ag 3d_3/2_ and Ag 3d_5/2_ peaks. The two main peaks at 572 and 582 eV may be attributed to the binding energies of Te 3d_3/2_ and Te 3d_5/2_, respectively, implying the Te^2-^ atomic state (Figure 5A). Additionally, two small Te peaks at 576 and 586 eV observed in Figure 5A indicate the presence of Te^4+^ oxide (Samal and Pradeep, 2009). Wang et al. (Wang et al., 2019) compared the Te 3d_5/2_ peaks of n-type and p-type Te/Ag_2_Te. They observed a notable difference in the spectra for the n-type compound at 571 and p-type compound at 573. The results of this study confirmed that the film consists of p-type Ag_2_Te. Over time, the oxide is formed owing to the slight oxidation of Te NWs in the atmosphere during the formation of Ag_2_Te. The considerably low intensity of the peaks suggests that with time, Te is converted to Ag_2_Te.

In XPS, the peak position indicates the elemental and chemical composition, whereas the peak intensity indicates the amount present on the material surface (Shard et al., 2019; Watts and Wolstenholme, 2019). As shown in Figure 5B, the intensity of the peak corresponding to Ag increases with the reaction time. This further confirms that Ag doping in Te NWs results in an increase in the reaction time. The electrical conductivity of the Te NWs is 0.11 S/cm, and the electrical conductivities at reaction times of 3, 5, 7, and 10 min are 1.2, 1.52, 1.69, and 0.016 S/cm, respectively, as shown in Table 1. When the topotactic reaction was performed for 7 min, the highest electrical conductivity was achieved at 1.69 S/cm, which is approximately 15 times higher than that of undoped Te NWs. This further indicates that electrical conductivity increases according to the Ag content of Te NWs as the intensity of the peak corresponding to Ag increases. However, after the 10 min reaction, Ag-Te exhibits low electrical conductivity, which can be attributed to the breaking of wires due to the brittleness of Ag_2_Te. This suggests that the highest electrical conductivity can be achieved at an optimal Ag doping level and reaction time of 7 min, beyond which the structural integrity of the nanowires is compromised.

**TABLE 1:** Electrical conductivity of the Te and Ag-Te samples.

Materials	Te NWs	Ag-Te NWs (3 min)	Ag-Te NWs (5 min)	Ag-Te NWs (7 min)	Ag-Te NWs (10 min)
Electrical conductivity (S/cm)	0.11	1.20	1.52	1.69	0.0160

**FIGURE 6:**
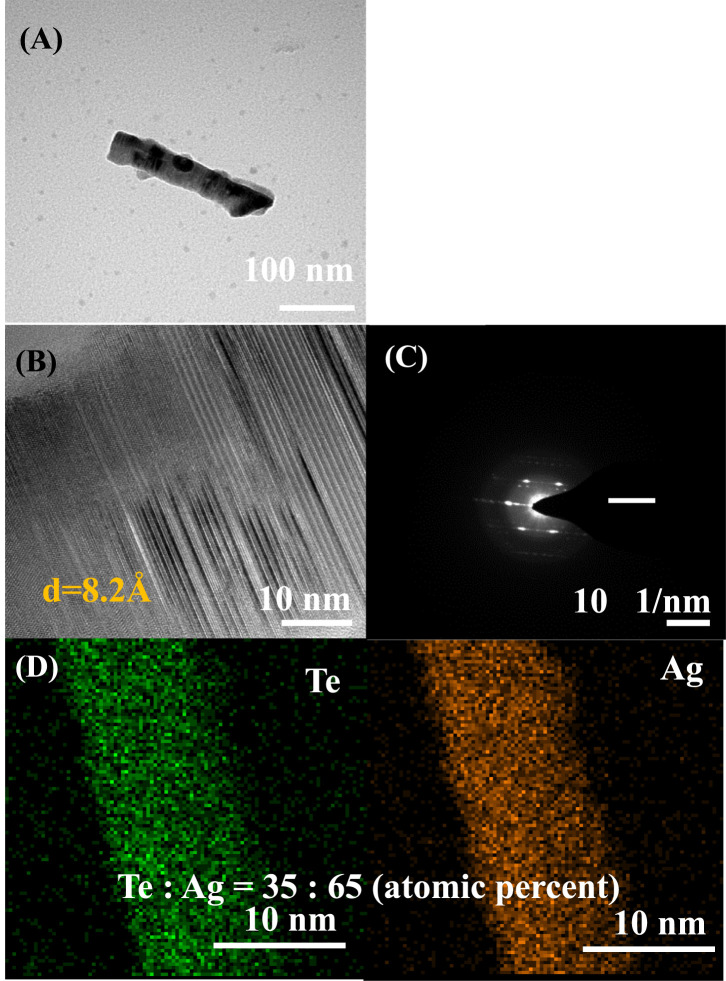
**(A)** TEM image **(B)** HR-TEM image **(C)** SAED pattern **(D)** EDX mapping profile of Ag-Te NWs (reaction time: 7 min).

Figure 6 shows the structure, morphology, and elemental ratio of the Ag-Te NWs synthesized under optimized conditions determined through TEM, SAED, and EDX. Figure 6A shows the TEM image of the Ag-Te NWs. The interplanar distance is 8.2 Å, which corresponds to the (010) lattice plane of Ag_2_Te (Figure 6B). Moreover, background interference is detected in the XRD patterns, and the HR-TEM image and SAED pattern indicate the formation of polycrystalline structures rather than single crystals (Figures 6B, C, respectively). Figure 6D shows the EDX map of the Ag-TE NWs synthesized through the topotactic reaction in this study. This reaction transforms Te to Ag-Te, starting from the surface, which can lead to irregularities in crystal formation. Despite these observations, the feature observed in the SAED patterns (Macmillan et al., 2016), described as blurring due to structural transitions as well as the XRD patterns suggest that the synthesis results in a composite material containing both Te and Ag_2_Te phases. The reason for the observed XRD noise is likely the small sample area, because the Te NWs have a diameter of 100 nm, limiting the observable area. According to the EAS Department - University of Alberta, small sample sizes can significantly impact the XRD results, introducing noise and affecting the quality of the diffraction patterns.

**FIGURE 7:**
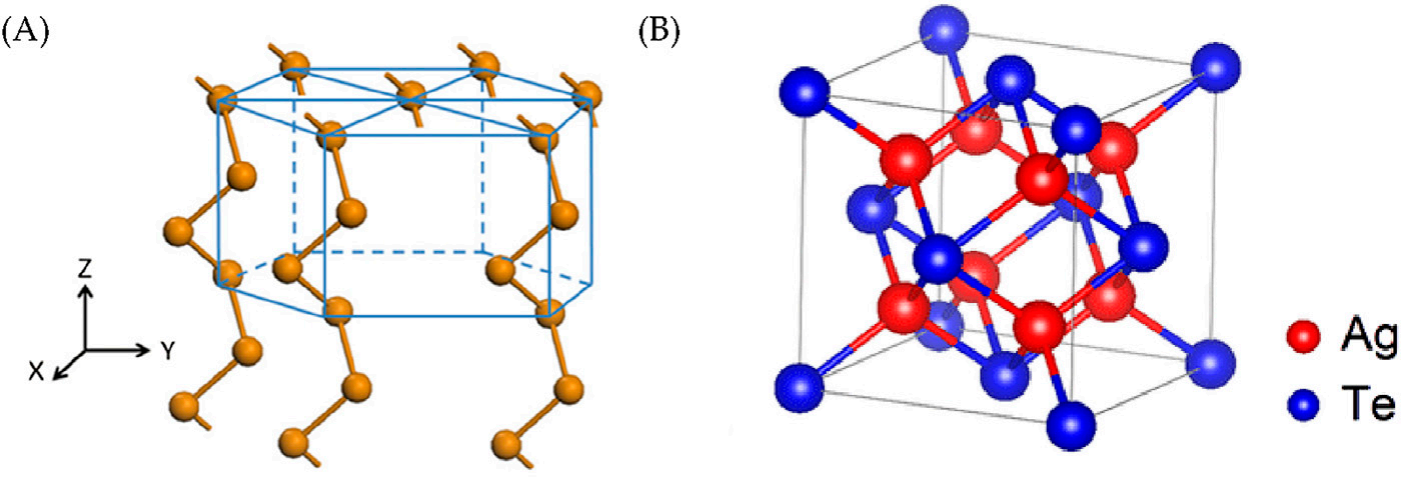
Crystal structures of the **(A)** Te and **(B)** Ag-Te atoms.

A transition occurs as the Ag loading increases, and the Te (010) plane (as shown in Figure 7A) (Chen and Qin, 2020) experiences lattice stretching and subsequently transforms to the hexagonal Ag_5_Te_3_ structure. At a higher Ag loading, a second transition occurs, and a Ag_2_Te structure is obtained. During this process, Ag adopts a face-centered cubic arrangement, as depicted in Figure 7B (Wu et al., 2018). Specifically, the transitions are observed at Ag loading values of 10% for Ag_5_Te_3_ and 25% for Ag_2_Te.

**FIGURE 8:**
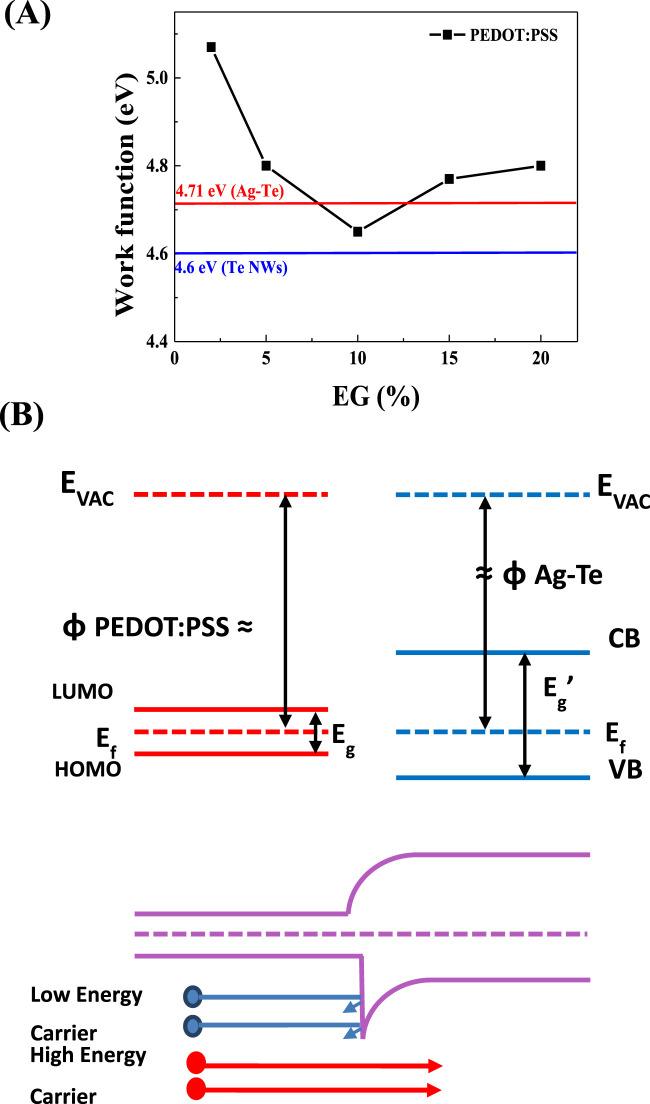
**(A)** Work function of the Te NWs prepared using 2, 5, 10, 15, 20, 25% of EG with PEDOT:PSS **(B)** Schematic illustrating how the presence of a suitable energy barrier can result in energy filtering.

To improve the thermoelectric performance of the synthesized Ag-Te NWs, an organic–inorganic composite (Ag-Te NWs/PEDOT:PSS) was synthesized. To synthesize this hybrid thermoelectric material, doping was performed using PEDOT:PSS. Figure 8A shows the work functions of PEDOT:PSS at different EG concentrations: 2%, 5%, 10%, 15%, and 20%, which were determined to be 5.07, 4.8, 4.65, 4.77, and 4.8 eV, respectively. The determined work functions of the Te NWs and Ag-Te NWs are 4.6 and 4.71 eV, respectively. At 15% EG, the energy barrier is expected to be at 0.06 eV, indicating improvement in the thermoelectric performance due to the energy filtering effect. Figure 8B shows a schematic of the energy filtering effect between PEDOT:PSS and Ag-Te NWs. An appropriate interfacial energy barrier in the nanocomposite can help improve the Seebeck coefficient by impeding the movement of low-energy carriers and promoting the movement of high-energy carriers. The theoretical effective energy barrier to maximize the work function is less than 0.2 eV (Zhang et al., 2010; Song and Cai, 2017; Liang et al., 2017). The improved thermoelectric performance is evidenced by a Seebeck coefficient of 69.5 μV/K and a PF of 260 μW/mK^2^.

**FIGURE 9:**
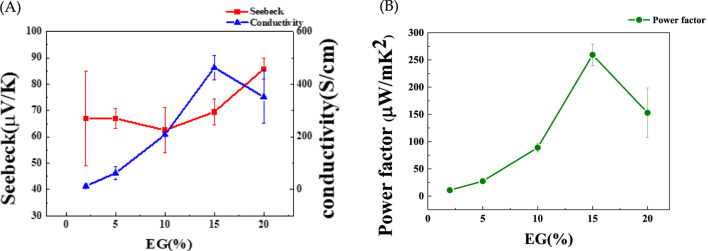
**(A)** Seebeck coefficient, electrical conductivity, and **(B)** power factor (PF) of Ag-Te NWs/PEDOT:PSS at varying EG concentrations.

In this study, we investigated the influence of the PSS content of PEDOT:PSS on the thermoelectric properties of the Ag-Te NWs by adjusting the EG concentration. The EG concentrations of 2%, 5%, 10%, 15%, and 20% were chosen and a consistent reaction time of 7 min was maintained for all samples. As shown in Figure 9A, the electrical conductivities are 13, 62, 208, 463, and 351 S/cm, with the corresponding Seebeck coefficients of 66.9, 67, 62.5, 69.5, and 85.8 μV/K. The PF peaks at 260 μW/mK^2^ at 15% EG (Figure 9B), indicating a significant improvement in the electrical conductivity (463 S/cm) in relation to that of untreated Ag-Te NWs (1.69 S/cm). The improvement in the conductivity can be largely attributed to the increased electrical conductivity of PEDOT:PSS (810 S/cm (Wei et al., 2016)), which compensates for the inherently low conductivity of Te.

Furthermore, the introduction of an energy filtering system at the interface between the Ag-Te NWs and PEDOT:PSS creates an optimal energy barrier of 0.06 eV at 15% EG, significantly enhancing the Seebeck coefficient through the selective blocking of low-energy carriers (Figure 9A). In energy filtering, both the low- and high-energy carriers tend to accumulate at the colder end of the material. The optimal energy barrier in the thermoelectric material effectively filters the low-energy holes near the Fermi level, which results in an increase in the average energy of carriers. However, balancing the height of this barrier is crucial; an excessively high barrier may hinder the movement of both the high- and low-energy carriers, further diminishing the overall conductivity of the material (Shi et al., 2020; Liang et al., 2017; Singh and Ahuja, 2022).

The variations in the electrical conductivity were observed to correlate with the EG content, which was instrumental in adjusting the PSS content of PEDOT:PSS. Pristine PEDOT:PSS exhibits low electrical conductivity owing to the existence of insulating PSS molecules, which enable the dispersant complex to form films (Wei et al., 2020). Therefore, EG can facilitate the formation of PSS chains and the optimization of π–π-stacking distances, resulting in an increase in electrical conductivity.

Our research also investigated the structural features of organic thermoelectric materials, with crystal sizes ranging from nanometers to micrometers. The specific arrangement of these crystals has a considerable impact on the electrical conductivity. In semicrystalline conductive polymers, the electronic band structure can be tuned through macromolecular engineering and further refined via adequate structuring into highly ordered crystalline regions (Prunet et al., 2021). This refinement process, known as secondary doping, significantly enhances the electronic properties of polymers, highlighting the influence of structural control on the performance of organic thermoelectric materials.

Increased crystallinity facilitates smoother charge transport, thus improving electrical conductivity. The treatment of a conductive polymer film with solvents, whether in a liquid or vapor form, alters its morphology without affecting the doping level (Prunet et al., 2021; Kirchmeyer and Reuter, 2005). At a microscale, the bonding interactions between molecules and polymers may result in varied modes of electrical charge transport within each crystal. Techniques such as mechanical stretching and the use of specific solvents can optimize molecular alignment, which may enhance bonding and facilitate electrical charge transport. A notable molecular-level phenomenon is 'π–π stacking,’ wherein strong intermolecular bonds are formed. Stacking plays a crucial role in the introduction and movement of electrical charges between molecules, which is critical for the functionality of these materials (Zhang et al., 2010; Russ et al., 2016; Song and Cai, 2017; Lee et al., 2020).

Table 2 lists the thermoelectric properties of different Te composites synthesized in various forms. Remarkably, the PF of the as-prepared Ag-Te NWs/PEDOT:PSS composite, recorded at 260 μW/mK2, surpasses that of previously reported systems (Meng et al., 2019). Note that the configurations detailed in Table 2 are based on randomly arranged composites. In contrast to the materials listed in the table, the present study focuses on aligned configurations, allowing the mor precise control of the properties of the synthesized materials. This result not only underscores the higher electrical conductivity of the as-prepared composite than that of other Te variants but also highlights the potential contribution of our synthesized materials toward further advances in thermoelectric technology.

**TABLE 2:** Thermoelectric properties of Te-based thermoelectric materials.

Materials	Form	σ (S/cm)	S (μV/K)	S2σ (μW/mK2)	Ref.
p-Ag2Te	NWs	0.110	213	0.499	Samal and Pradeep (2009)
p-type Ag-doped Te	NWs	0.430	356	5.46	Wang et al. (2019)
PEDOT:PSS-coated Te	Film	58.9	139	51.6	Meng et al. (2019)
Te nanorod	Nanorod	0.0460	367	0.620	Song and Cai (2017)
Te nanorod/PEDOT:PSS	Nanorod	11.5	151	26.2	Song and Cai (2017)
Ag-Te NWs/PEDOT:PSS	NWs	463	69.5	224	This work

## 4 Conclusion

In this study, an organic–inorganic composite thermoelectric material, Ag-Te NWs/PEDOT:PSS, was synthesized using aligned NWs. Through the topotactic reaction, high electrical conductivity was imparted to the composite by doping high-electrical-conductivity Ag to Te NWs, and a composite with the desired ratio was formed through a relatively simple experiment. In addition, a thermoelectric material with high PF was fabricated by increasing the electrical conductivity, which was achieved through the application of a conductive polymer to an inorganic material with a high Seebeck coefficient. Ag-Te NWs/PEDOT:PSS was doped to synthesize a composite with high thermoelectric performance because of the existence of high-energy carriers formed by the energy filtering effect. The optimized Ag-Te NWs/PEDOT:PSS composite exhibited an electrical conductivity of 463 S/cm, a Seebeck coefficient of 69.5 μV/K, and a PF of 260 μW/mK^2^ at 300 K. This PF value was higher than that of Te NWs/PEDOT:PSS (70.9 μW/mK^2^) by a factor of 3.6 (Yee et al., 2013); this increase was attributed to the synergistic effect of the two components. The fabrication of hybrid thermoelectric materials using aligned NWs presents a significant advancement in energy conversion technology. With enhanced conductivity and Seebeck coefficient, these materials hold promise for applications in waste heat recovery systems, portable electronics, and automotive exhaust systems, offering efficient conversion of heat to electricity. This research paves the way for more sustainable energy solutions by improving the performance of thermoelectric devices, contributing to the mitigation of climate change and the transition toward greener energy sources.

## Data Availability

The original contributions presented in the study are included in the article/supplementary material, further inquiries can be directed to the corresponding author.

## References

[B1] Bell L. E. (2008). Cooling, heating, generating power, and recovering waste heat with thermoelectric systems. Science 321 (5895), 1457–1461. 10.1126/science.1158899 18787160

[B2] Carraro C. Maboudian R. Magagnin L. (2007). Metallization and nanostructuring of semiconductor surfaces by galvanic displacement processes. Surf. Sci. Rep. 62 (12), 499–525. 10.1016/j.surfrep.2007.08.002

[B3] Chen R. Lee J. Lee W. Li D. (2019). Thermoelectrics of nanowires. Chem. Rev. 119 (15), 9260–9302. 10.1021/acs.chemrev.8b00627 30882214

[B4] Chen R. Xu D. Guo G. Gui L. (2002). Silver telluride nanowires prepared by dc electrodeposition in porous anodic alumina templates. J. Mater. Chem. 12 (8), 2435–2438. 10.1039/b201007k

[B5] Chen Z.-Y. Qin R. (2020). Probing structural chirality of crystals using high-order harmonic generation in solids. Phys. Rev. A 101 (5), 053423. 10.1103/physreva.101.053423

[B6] Floro J. A. Chason E. Cammarata R. C. Srolovitz D. J. (2002). Physical origins of intrinsic stresses in volmer–weber thin films. M.R.S. Bull. 27 (1), 19–25. 10.1557/mrs2002.15

[B7] Gao Z. Liu G. Ren J. (2018). High thermoelectric performance in two-dimensional tellurium: an *ab initio* study. A.C.S. Appl. Mat. Interfaces 10 (47), 40702–40709. 10.1021/acsami.8b11836 30394087

[B8] Gnanadurai P. Soundararajan N. Sooriamoorthy C. E. (2002). Investigation on the influence of thickness and temperature on the Seebeck coefficient of silver telluride thin films. Vacuum 67 (2), 275–284. 10.1016/s0042-207x(02)00274-9

[B9] Goldsmid H. J. Gray A. S. (1979). Thermoelectric refrigeration at very low temperatures. Cryogenics 19 (5), 289–292. 10.1016/0011-2275(79)90146-2

[B10] Hangarter C. M. Myung N. V. (2005). Magnetic alignment of nanowires. Chem. Mat. 17 (6), 1320–1324. 10.1021/cm047955r

[B11] Hasan M. N. Nayan N. Nafea M. Muthalif A. G. A. Mohamed Ali M. S. (2022). Novel structural design of wearable thermoelectric generator with vertically oriented thermoelements. Energy 259, 125032. 10.1016/j.energy.2022.125032

[B12] Ishiwata S. Shiomi Y. Lee J. S. Bahramy M. S. Suzuki T. Uchida M. (2013). Extremely high electron mobility in a phonon-glass semimetal. Nat. Mat. 12 (6), 512–517. 10.1038/nmat3621 23603851

[B13] Jeong D.-B. Lim J.-H. Lee J. Park H. Zhang M. Lee Y.-I. (2013). Template-free synthesis of vertically oriented tellurium nanowires via a galvanic displacement reaction. Electrochim. Acta 111, 200–205. 10.1016/j.electacta.2013.07.228

[B14] Jung H. Rheem Y. Chartuprayoon N. Lim J.-H. Lee K.-H. Yoo B. (2010). Ultra-long bismuth telluride nanoribbons synthesis by lithographically patterned galvanic displacement. J. Mat. Chem. 20 (44), 9982. 10.1039/c0jm02058c

[B15] Kim G. H. Shao L. Zhang K. Pipe K. P. (2013). Engineered doping of organic semiconductors for enhanced thermoelectric efficiency. Nat. Mat. 12 (8), 719–723. 10.1038/nmat3635 23644522

[B16] Kim J. Zhang M. Bosze W. Park S. D. Lim J. H. Myung N. V. (2015). Maximizing thermoelectric properties by nanoinclusion of γ-SbTe in Sb_2_Te_3_ film via solid-state phase transition from amorphous Sb–Te electrodeposits. Nano Energy 13, 727–734. 10.1016/j.nanoen.2015.03.020

[B17] Kirchmeyer S. Reuter K. (2005). Scientific importance, properties and growing applications of poly(3,4-ethylenedioxythiophene). J. Mat. Chem. 15 (21), 2077. 10.1039/b417803n

[B18] Kok K.-Y. Choo T.-F. Saidin N. U. Che Ab Rahman C. Z. C. (2018). Large-scale synthesis of tellurium nanostructures via galvanic displacement of metals. I.O.P. Conf. Ser. Mat. Sci. Eng. 298, 012015. 10.1088/1757-899x/298/1/012015

[B19] Lee S. Kim S. Pathak A. Tripathi A. Qiao T. Lee Y. (2020). Recent progress in organic thermoelectric materials and devices. Macromol. Res. 28 (6), 531–552. 10.1007/s13233-020-8116-y

[B20] Liang Z. Boland M. J. Butrouna K. Strachan D. R. Graham K. R. (2017). Increased power factors of organic–inorganic nanocomposite thermoelectric materials and the role of energy filtering. J. Mat. Chem. A 5 (30), 15891–15900. 10.1039/c7ta02307c

[B21] Lim J. H. Shin G. J. Hwang T. Y. Lim H. R. Lee Y. I. Lee K. H. (2014). Three-dimensional hierarchical Te–Si nanostructures. Nanoscale 6 (20), 11697–11702. 10.1039/c4nr02469a 24988904

[B22] Lin S. Li W. Chen Z. Shen J. Ge B. Pei Y. (2016). Tellurium as a high-performance elemental thermoelectric. Nat. Commun. 7, 10287. 10.1038/ncomms10287 26751919 PMC4729895

[B23] Macmillan E. Ciobanu C. L. Ehrig K. Cook N. J. Pring A. (2016). Replacement of uraninite by bornite via coupled dissolution-reprecipitation: evidence from texture and microstructure. Can. Mineral. 54 (6), 1369–1383. 10.3749/canmin.1600031

[B24] Mao J. Liu Z. Ren Z. (2016). Size effect in thermoelectric materials. npj Quant. Mat. 1 (1), 16028. 10.1038/npjquantmats.2016.28

[B25] Mazzio K. A. Kojda D. Rubio-Govea R. Niederhausen J. Ryll B. Raja-Thulasimani M. (2020). P-Type-to-N-Type transition in hybrid Ag_x_Te/PEDOT:PSS thermoelectric materials via stoichiometric control during solution-based synthesis. A.C.S. Appl. Energy Mat. 3 (11), 10734–10743. 10.1021/acsaem.0c01774

[B26] Meng Q. Jiang Q. Cai K. Chen L. (2019). Preparation and thermoelectric properties of PEDOT:PSS coated te nanorod/PEDOT:PSS composite films. Org. Electron. 64, 79–85. 10.1016/j.orgel.2018.10.010

[B27] Mohanty P. Kang T. Kim B. Park J. (2006). Synthesis of single crystalline tellurium nanotubes with triangular and hexagonal cross sections. J. Phys. Chem. B 110 (2), 791–795. 10.1021/jp0551364 16471604

[B28] Moon G. D. Ko S. Min Y. Zeng J. Xia Y. Jeong U. (2011). Chemical transformations of nanostructured materials. Nano Today 6 (2), 186–203. 10.1016/j.nantod.2011.02.006

[B29] Park H. Jung H. Zhang M. Chang C. H. Ndifor-Angwafor N. G. Choa Y. (2013). Branched tellurium hollow nanofibers by galvanic displacement reaction and their sensing performance toward nitrogen dioxide. Nanoscale 5 (7), 3058–3062. 10.1039/c3nr00060e 23463030

[B30] Prunet G. Pawula F. Fleury G. Cloutet E. Robinson A. J. Hadziioannou G. (2021). A review on conductive polymers and their hybrids for flexible and wearable thermoelectric applications. Mat. Today Phys. 18, 100402. 10.1016/j.mtphys.2021.100402

[B31] Rudnik E. Sobesto J. (2011). Cyclic voltammetric studies of tellurium in diluted HNO3 solutions. Archives Metallurgy Mater. 56 (2), 270–277. 10.2478/v10172-011-0030-z

[B32] Russ B. Glaudell A. Urban J. J. Chabinyc M. L. Segalman R. A. (2016). Organic thermoelectric materials for energy harvesting and temperature control. Nat. Rev. Mat. 1 (10), 16050. 10.1038/natrevmats.2016.50

[B33] Samal A. K. Pradeep T. (2009). Room-temperature chemical synthesis of silver telluride nanowires. J. Phys. Chem. C 113 (31), 13539–13544. 10.1021/jp901953f

[B34] Shard A. G. Counsell J. D. P. Cant D. J. H. Smith E. F. Navabpour P. Zhang X. Intensity calibration and sensitivity factors for XPS instruments with, 2019.

[B35] Shi H. Liu C. Jiang Q. Xu J. (2015). Effective approaches to improve the electrical conductivity of PEDOT:PSS: a review. Adv. Elect. Mat. 1 (4). 10.1002/aelm.201500017

[B36] Shi X. L. Zou J. Chen Z. G. (2020). Advanced thermoelectric design: from materials and structures to devices. Chem. Rev. 120 (15), 7399–7515. 10.1021/acs.chemrev.0c00026 32614171

[B37] Singh D. Ahuja R. (2022). Dimensionality effects in high‐performance thermoelectric materials: computational and experimental progress in energy harvesting applications. WIREs Comput. Mol. Sci. 12 (1). 10.1002/wcms.1547

[B38] Song H. Cai K. (2017). Preparation and properties of PEDOT:PSS/Te nanorod composite films for flexible thermoelectric power generator. Energy 125, 519–525. 10.1016/j.energy.2017.01.037

[B39] Toberer G. J. S. a.E. S. (2008). Complex thermoelectric materials. Nat. Mat. 7, 10–12. 10.1038/nmat2090 18219332

[B40] Wang Q. Safdar M. Wang Z. He J. (2013). Low-dimensional Te-based nanostructures. Adv. Mat. 25 (28), 3915–3921. 10.1002/adma.201301128 24048978

[B41] Wang W. Li C. Li X. Jia Y. Jiang F. Xu J. (2018). Fabrication of freestanding tellurium nanofilm and its thermoelectric performance. Thin Solid Films 654, 23–29. 10.1016/j.tsf.2018.03.073

[B42] Wang W. Liu J. Li X. Jiang Q. Xu J. Luo C. (2019). Galvanic exchange reaction involving te nanowires and Ag ions for n-type Te/Ag2Te thermoelectric nanofilms. J. Nanopart. Res. 21 (6), 131. 10.1007/s11051-019-4536-z

[B43] Watts J. F. Wolstenholme J. (2019). An introduction to surface analysis by XPS and AES. John Wiley and Sons.

[B44] Wei J. Yang L. Ma Z. Song P. Zhang M. Ma J. (2020). Review of current high-ZT thermoelectric materials. J. Mat. Sci. 55 (27), 12642–12704. 10.1007/s10853-020-04949-0

[B45] Wei Q. Uehara C. Mukaida M. Kirihara K. Ishida T. (2016). Measurement of in-plane thermal conductivity in polymer films. A.I.P. Adv. 6 (4). 10.1063/1.4948447

[B46] Wu B. Zhou Y. Hu M. (2018). Two-Channel thermal transport in ordered-disordered superionic Ag_2_Te and its traditionally contradictory enhancement by nanotwin boundary. J. Phys. Chem. Lett. 9 (19), 5704–5709. 10.1021/acs.jpclett.8b02542 30222358

[B47] Xiao F. Chen G. Wang Q. Wang L. Pei J. Zhou N. (2010). Simple synthesis of ultra-long Ag2Te nanowires through solvothermal Co-reduction method. J. Solid State Chem. 183 (10), 2382–2388. 10.1016/j.jssc.2010.07.020

[B48] Yee S. K. Coates N. E. Majumdar A. Urban J. J. Segalman R. A. (2013). Thermoelectric power factor optimization in PEDOT:PSS tellurium nanowire hybrid composites. Phys. Chem. Chem. Phys. 15 (11), 4024–4032. 10.1039/c3cp44558e 23400218

[B49] Yuan M. Sun L. Lu X. W. Jiang P. Bao X. H. (2021). Enhancing the thermoelectric performance of Cu–Ni alloys by introducing carbon nanotubes. Mat. Today Phys. 16, 100311. 10.1016/j.mtphys.2020.100311

[B50] Zeng X. Yan C. Ren L. Zhang T. Zhou F. Liang X. (2019). Silver telluride nanowire assembly for high-performance flexible thermoelectric film and its application in self-powered temperature sensor. Adv. Elect. Mat. 5 (2). 10.1002/aelm.201800612

[B51] Zhang B. Sun J. Katz H. E. Fang F. Opila R. L. (2010). Promising thermoelectric properties of commercial PEDOT:PSS materials and their bi2Te3 powder composites. A.C.S. Appl. Mat. Interfaces 2 (11), 3170–3178. 10.1021/am100654p 21053917

[B52] Zhang W. Yu R. Feng W. Yao Y. Weng H. Dai X. (2011). Topological aspect and quantum magnetoresistance of beta-Ag2Te. Phys. Rev. Lett. 106 (15), 156808. 10.1103/physrevlett.106.156808 21568599

